# Impact of COVID-19 Pandemic and Lockdown on the Epidemiology of RSV-Mediated Bronchiolitis: Experience from Our Centre

**DOI:** 10.3390/children9111723

**Published:** 2022-11-09

**Authors:** Sara Manti, Alessandro Giallongo, Giuseppe Fabio Parisi, Maria Papale, Santiago Presti, Manuela Lo Bianco, Lucia Spicuzza, Salvatore Leonardi

**Affiliations:** 1 Department of Clinical and Experimental Medicine, University of Catania, 95123 Catania, Italy; 2 Pediatric Unit, Ospedale Maggiore, 97015 Modica, Italy; 3 Respiratory Unit, A.O.U. Policlinico-Vittorio Emanuele, 95123 Catania, Italy

**Keywords:** RSV, bronchiolitis, epidemiology, respiratory infection, COVID-19, lockdown

## Abstract

Background: The COVID-19 pandemic has dramatically affected the global epidemiology of other infectious respiratory diseases, leading to a significant decrease in their incidence. Hence, we aimed to characterize the epidemiology of RSV-bronchiolitis in children. Methods: children aged ≤2 years diagnosed with RSV-mediated bronchiolitis admitted to our Unit from October 2018 to December 2021, were retrospectively enrolled. Results: We included 95 patients (M/F = 46/49; mean age 7.56 ± 6.6 months). Specifically, 17 infants in 2018, 34 in 2019, 0 during 2020 lockdown, 1 during 2020 post-lockdown, and 43 in 2021. Incidence was significantly lower in 2020 compared with 2018, 2019 and 2021 (*p* < 0.05). No differences were found concerning need for respiratory support. Discussion: Several factors related to SARS-CoV-2 pandemic, especially restrictive measures, may have contributed to a significant reduction in hospitalizations due to RSV. The new outbreak in RSV infection-related hospitalizations reported between October and December 2021 has been suggested it may be due to an increased number of susceptible individuals to RSV infection. Conclusion: The experience of the SARS-CoV-2 outbreak has led to a marked decrease in other viral respiratory infections, such as RSV. This may pave the way for new approaches in preventing respiratory infections, highlighting the role of preventive measures.

## 1. Introduction

The COVID-19 pandemic has dramatically affected not only the entire world and every aspect of life, but also the global epidemiology of diseases in the human population. Where on the one hand, the lockdown, social distancing, and other public health measures have increased the incidence of communicable and chronic diseases, such as cancer; on the other hand, the restrictive measures to curb the spread of SARS-CoV-2 have led to a significant decrease in the incidence of infectious respiratory diseases, such as influenza and parainfluenza viruses, metapneumovirus, rhinovirus, and respiratory syncytial virus (RSV) infection. Regarding the latter, it has been reported a significant reduction in hospitalization, up to 70%, for RSV-mediated bronchiolitis in infants younger than one year [1,2]. However, evidence on the new epidemiologic trend of RSV is lacking. Currently, it is impossible to foresee whether this is a transitory phenomenon or whether RSV can re-emerge following its old epidemiologic wave or with a new epidemiologic wave, maintaining the same virulence or being associated with more severe disease course and/or co-circulating with SARS-CoV-2. Accordingly, epidemiological data are needed to address these issues and help researchers and physicians in their clinical practice. In this regard, we aimed to assess the impact of the COVID-19 pandemic and the subsequently adopted measures to face the pandemic on RSV-mediated bronchiolitis disease severity and hospitalization rates in infants. A pilot clinical, retrospective, monocentric study was designed.

## 2. Materials and Methods

### 2.1. Subjects and Eligibility Criteria

We enrolled infants who fulfilled the criteria for RSV-mediated bronchiolitis diagnosis and who had been referred to the Paediatric Respiratory and Cystic Fibrosis Unit, San Marco Hospital, University of Catania, from October 2018 to December 2021.

Inclusion criteria: patients of both sexes, aged less than 2 years and diagnosed with bronchiolitis according to the current guidelines [3]; RSV infection assessed by reverse transcription polymerase chain reaction (RT-PCR) performed on nasopharyngeal swab specimen [4]; parents’ children have signed informed consent for the study. 

Exclusion criteria: children older than 2 years old; patients diagnosed with other infectious respiratory diseases (influenza virus, parainfluenza virus, rhinovirus, metapneumoviruses, adenovirus), anatomic malformation, any chronic diseases.

The institutional Review Board of the University of Catania approved the study (Prot. n. 10290 3 March 2021, n. 18/2021), and written informed consent was obtained from the parents’ patients [5].

### 2.2. Timing of the Study

In Italy, the COVID-19 lockdown started on 9 March 2020 and lasted until 19 May 2020. The study was structured in the following periods: before lockdown (T-1): from October 2018 to 8 March 2020; during lockdown (T0): from 9 March 2020 to 19 May 2020; and after lockdown (T1): from 20 May 2020 to 31 December 2021.

## 3. Results

**Figure 1:**
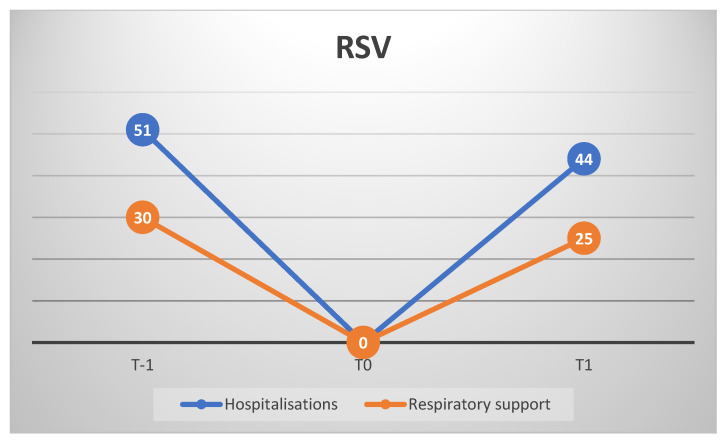
Hospitalized patients for RSV-mediated bronchiolitis (blue line) and patients who needed respiratory support (orange line). T-1: 2018–2019; T0 2020 lockdown; T1: 2020 post-lockdown and 2021.

Demographic and clinical data of the enrolled infants were retrospectively analysed. We included 95 patients (M/F = 46/49; mean age 7.56 ± 6.6 months) affected by RSV-induced bronchiolitis between 2018 and 2021. Of these, 51 at T-1, 0 at T0, and 44 at T1. Specifically, 17 babies in 2018, 34 in 2019, 0 during 2020 lockdown, 1 during 2020 post-lockdown, and 43 in 2021. Incidence was significantly lower in 2020 compared with 2018, 2019, and 2021 (*p* < 0.001). If needed, a respiratory support was provided, consisting of high flow nasal cannula (HFNC) at first, and helmet continuous positive airway pressure (CPAP), in the event of no improvement with HFNC. In this regard, 14 infants out of 17 needed respiratory support (HFNC:CPAP = 13:1) in 2018, 16/34 (HFNC:CPAP = 12:4) in 2019, 1/1 (HFNC:CPAP = 0:1) in 2020 post-lockdown, and 25/43 (HFNC:CPAP = 21:4) in 2021. Notably, in 2021, 40 hospitalisations out of 43 were reported between the end of October and December. The percentage of severe cases was not statistically significant in 2020–2021 compared with 2018–2019 (*p* > 0.05) (Figure 1).

## 4. Discussion

We reported a significant reduction in hospitalisations due to RSV infection during the SARS-CoV-2 pandemic in a tertiary centre of Southern Italy, followed by a resurge at the end of 2021.

### 4.1. Pandemic Health Protections

Our data are consistent with those of another tertiary centre in northern Italy, which reported a significant drop in hospitalisations for bronchiolitis, suggesting that infants have indirectly benefited from measures to contain the SARS-CoV2 pandemic and school closure [6,7]. Midulla et al. have also reported this tendency worldwide, which may probably be due to the restrictive measures introduced to face SARS-CoV-2 spread, such as face masks, hand hygiene, social distancing, and lockdown [8]. 

Before the SARS-CoV-2 pandemic, a meta-analysis of 51 studies reported that RSV was the most common aetiology of bronchiolitis in children aged less than 2 years (59.2%; 95% CI 54.7–63.6) [9]. RSV infection represented 19–81% of hospitalisation for acute respiratory infections, and the rate of hospital admissions was inversely correlated with lower age (75–90% of infants were aged less than 1 year old). Such a wide range in hospitalization associated with RSV infection among studies could be due to reduced test sensitivity in older studies [10]. A systematic review reported global incidence of RSV infection-associated hospitalisation was 19.19 per 1000 children per year (95% CrI, 15.04–24.48) among children aged <1 year, and this was higher if they were preterm 63.85 (95% CrI, 37.52–109.7) [11]. Surprisingly, 2020 was characterised by a significant fall (>99%) in RSV cases detected [12].

Since RSV transmission occurs through aerosol, similar to SARS-CoV-2, it has been suggested that restrictive measures, in effect also in our country, have significantly impacted on the disappearance of RSV epidemy [8,13]. In their systematic review, authors showed that the implemented public health and social measures to control the spread of SARS-CoV-2 were associated with an overall decrease of 23–94% in the incidence of respiratory viral diseases and a decrease of 0–98% in the detection of the viruses [14].

### 4.2. Environmental Considerations

Environmental conditions, both weather and air pollution, also affect the incidence of RSV. Though climate factors influence the spread of RSV infection, their contribution is variable according to the different areas of the world. Indeed, RSV transmission is positively correlated with lower temperatures and higher relative humidity rates. Climate conditions, such as rain that made people stay home and crowded places, are associated with increased RSV infection rates [15]. A ten-year study on Italian infants aged less than 12 months reported RSV-induced bronchiolitis following winter seasonality with a peak between December and February. The authors found a correlation between RSV–bronchiolitis incidence, benzene level, and relative humidity, while an inverse correlation with temperature was detected [16]. Indeed, in the northern hemisphere regions with a temperate climate, the spread of RSV infection is typically seasonal during winter [17]. Conversely, this trend did not occur during the 2020 lockdown and winter 2020–2021 in Belgium and New Zealand, where the reduction in RSV cases was around 98–99% compared with the same period of the previous year (2019) [12,18]. Data from our centre are consistent with the two aforementioned studies. Though the small sample, after lockdown, only one case of a patient with severe RSV bronchiolitis was observed during the summer 2020, out of the usual RSV seasonality, despite the hot weather of Sicily. 

As with regard to air pollutants (benzene and nitrogen oxides), a significant decrease, up to 60%, was registered in the Catania area, as in the rest of Italy, during the 2020 lockdown. Therefore, a role of reduced air pollution in the epidemiology of RSV could be speculated, though it is hard to quantify its contribution; we suggest, however, that it is minor if compared with restrictive measures [19]. 

### 4.3. Transmission in Schools

A trend in delayed or missed RSV epidemics was observed worldwide after the first COVID-19 pandemic wave, and school re-opening was one of the non-pharmaceutical interventions associated with increased RSV circulation [20]. Contrary to this other than our results, France reported an RSV epidemic during the winter 2020–2021, just after the end of the lockdown. However, during the lockdown, schools remained open in France, and the epidemic had a temporal association with the release of restrictions among adults, which may have contributed to the spread of RSV infection. Furthermore, in France, the incidence of severe cases reduced, probably because median age of infected children in 2020/2021 was higher than in previous years [21] and the epidemic of bronchiolitis reached a peak in March 2021, later than in previous years [22]. These findings were consistent with data from Finland and Spain, where school re-opening was not associated with an increase in the rate of RSV hospitalization, and a delayed peak was observed in July 2021 [23,24]. Hence, an unexpected role of adults in RSV transmission or as RSV reservoir has been suggested [25]. This is in part consistent with our data, since the Italian government reintroduced restrictive measures to curb a surge in COVID-19 cases at the end of November 2020, and we did not report RSV infection-related hospitalizations, despite elementary schools and kindergartens had been opened since September 2020.

### 4.4. Increased Susceptibility to RSV: “The Immunity Debt”

A rise in RSV cases was also reported in the United Kingdom in July 2021, out of its typical seasonality, among children aged 1–3 years. Median age of patients was higher than the pre-pandemic data. The authors have suggested a role of immunity duration, which decreased during the time and without exposure to RSV [26]. Even pregnant women could have had reduced exposure to RSV, whose infection, especially during the third trimester of pregnancy, could protect new-borns and infants against RSV infection through transplacental transfer of antibodies, antibodies synthesized by the fetus, and antibodies contained in breast milk [27,28,29]. Lung microbiota composition may also play a role in the susceptibility to viral infections [30]. The previously mentioned factors could increase the population of susceptible individuals and lead to other respiratory viruses’ epidemics, such as RSV [31]. The peak in RSV-related hospitalisations observed in Norway in October 2021 and the RSV outbreak occurred in Italy during the same period seems to support such a hypothesis [32,33]. In our centre, 41 RSV infection-related hospitalisations were reported between October and December 2021. However, the reappearance of RSV was not associated with a higher percentage of severe cases compared with the years before lockdown, 2019 and 2018.

### 4.5. Viral Infections and Wheezing

What we observed for RSV infection during lockdown also occurred in preschool children with recurrent wheezing, who experienced a significant reduction in medication use and respiratory exacerbations during lockdown, as a possible consequence of social distancing and reduced viral transmission [34].

## 5. Conclusions

We suggest that restrictions due to the SARS-CoV-2 pandemic significantly impacted on the reduced transmission of RSV and other respiratory tract infections. However, additional factors may have contributed to this, including the reduction in medical assistance being sought, due to the fear of COVID-19 infection, as shown by the significant drop in emergency department visits [35]. That may have led people to adopt a “wait and see” strategy for milder cases. On the other hand, severity disease was similar at presentation during COVID lockdowns and pre/post lockdown, suggesting that although mild cases presenting to the ED may have decreased, patients suffering from moderate-to-severe disease would still attend and for hospital admission be admitted.

Moreover, the efforts focused on testing for SARS-CoV-2 may have masked RSV infections. The influence of climate factors may be less than previously thought, whereas the percentage of RSV-naive people may be more significant. The experience of measures adopted during the SARS-CoV-2 outbreak may pave the way to a new approach in preventing respiratory infections, highlighting the role of preventive measures, such as hygiene measures and early detection and isolation of infected people, due to widespread PCR testing. National data centres are needed to monitor RSV epidemics to plan and reinforce these measures [36]. Non-pharmaceutical interventions combined with the introduction of vaccination and/or monoclonal antibodies other than palivizumab, which are in the pipeline, should aim at decreasing the burden of RSV infection, health-care-associated costs, and improve children’s outcomes [37,38,39]. Indeed, evidence shows that early acute viral infections in infancy might be associated with reduced lung function later in life, though other factors can contribute, among these the prematurity [40].

## Data Availability

The datasets used and/or analysed during the current study are available from the corresponding author on reasonable request.

## References

[1] Friedrich F., Ongaratto R., Scotta M.C., Veras T.N., Stein R.T., Lumertz M.S., Jones M.H., Comaru T., Pinto L.A. (2021). Early Impact of Social Distancing in Response to Coronavirus Disease 2019 on Hospitalizations for Acute Bronchiolitis in Infants in Brazil. Clin. Infect. Dis..

[2] Baraldi E., Lanari M., Manzoni P., Rossi G.A., Vandini S., Rimini A., Romagnoli C., Colonna P., Biondi A., Biban P. (2014). Inter-society consensus document on treatment and prevention of bronchiolitis in newborns and infants. Ital. J. Pediatr..

[3] RRalston S.L., Lieberthal A.S., Meissner H.C., Alverson B.K., Baley J.E., Gadomski A.M., Johnson D.W., Light M.J., Maraqa N.F., Mendonca E.A. (2014). Clinical practice guideline: The diagnosis, management, and prevention of bronchiolitis [published correction appears in Pediatrics. 2015 Oct;136(4):782]. Pediatrics.

[4] Heikkinen T., Marttila J., Salmi A.A., Ruuskanen O. (2002). Nasal swab versus nasopharyngeal aspirate for isolation of respiratory viruses. J. Clin. Microbiol..

[5] Manti S., Licari A. (2018). How to obtain informed consent for research. Breathe.

[6] Stera G., Pierantoni L., Masetti R., Leardini D., Biagi C., Buonsenso D., Pession A., Lanari M. (2021). Impact of SARS-CoV-2 Pandemic on Bronchiolitis Hospitalizations: The Experience of an Italian Tertiary Center. Children.

[7] Angoulvant F., Ouldali N., Yang D.D., Filser M., Gajdos V., Rybak A., Guedj R., Soussan-Banini V., Basmaci R., Lefevre-Utile A. (2021). Coronavirus Disease 2019 Pandemic: Impact Caused by School Closure and National Lockdown on Pediatric Visits and Admissions for Viral and Nonviral Infections—A Time Series Analysis. Clin. Infect. Dis..

[8] Di Mattia G., Nenna R., Mancino E., Rizzo V., Pierangeli A., Villani A., Midulla F. (2021). During the COVID-19 pandemic where has respiratory syncytial virus gone?. Pediatr. Pulmonol..

[9] Kenmoe S., Kengne-Nde C., Ebogo-Belobo J.T., Mbaga D.S., Fatawou Modiyinji A., Njouom R. (2020). Systematic review and meta-analysis of the prevalence of common respiratory viruses in children <2 years with bronchiolitis in the pre-COVID-19 pandemic era. PLoS ONE.

[10] Bont L., Checchia P.A., Fauroux B., Figueras-Aloy J., Manzoni P., Paes B., Simões E.A.F., Carbonell-Estrany X. (2016). Defining the Epidemiology and Burden of Severe Respiratory Syncytial Virus Infection Among Infants and Children in Western Countries. Infect. Dis. Ther..

[11] Stein R.T., Bont L.J., Zar H., Polack F.P., Park C., Claxton A., Borok G., Butylkova Y., Wegzyn C. (2017). Respiratory syncytial virus hospitalization and mortality: Systematic review and meta-analysis. Pediatr. Pulmonol..

[12] Van Brusselen D., De Troeyer K., Ter Haar E., Vander Auwera A., Poschet K., Van Nuijs S., Bael A., Stobbelaar K., Verhulst S., Van Herendael B. (2021). Bronchiolitis in COVID-19 times: A nearly absent disease?. Eur. J. Pediatr..

[13] Kulkarni H., Smith C.M., Lee D.D.H., Hirst R.A., Easton A.J., O’Callaghan C. (2016). Evidence of Respiratory Syncytial Virus Spread by Aerosol. Time to Revisit Infection Control Strategies?. Am. J. Respir. Crit. Care Med..

[14] Achangwa C., Park H., Ryu S., Lee M.S. (2022). Collateral Impact of Public Health and Social Measures on Respiratory Virus Activity during the COVID-19 Pandemic 2020–2021. Viruses.

[15] Tang J.W., Loh T.P. (2014). Correlations between climate factors and incidence—A contributor to RSV seasonality. Rev. Med. Virol..

[16] Nenna R., Evangelisti M., Frassanito A., Scagnolari C., Pierangeli A., Antonelli G., Nicolai A., Arima S., Moretti C., Papoff P. (2017). Respiratory syncytial virus bronchiolitis, weather conditions and air pollution in an Italian urban area: An observational study. Environ. Res..

[17] Borchers A.T., Chang C., Gershwin M.E., Gershwin L.J. (2013). Respiratory syncytial virus—A comprehensive review. Clin. Rev. Allergy Immunol..

[18] Huang Q.S., Wood T., Jelley L., Jennings T., Jefferies S., Daniells K., Nesdale A., Dowell T., Turner N., Campbell-Stokes P. (2021). Impact of the COVID-19 nonpharmaceutical interventions on influenza and other respiratory viral infections in New Zealand. Nat. Commun..

[19] QUALITA-DELLARIA-E-LOCKDOWN. https://www.snpambiente.it/wp-content/uploads/2020/12/QUALITA-DELLARIA-E-LOCKDOWN.pdf.

[20] Billard M., van de Ven P.M., Baraldi B., Kragten-Tabatabaie L., Bont L.J., Wildenbeest J.G. (2022). International changes in respiratory syncytial virus (RSV) epidemiology during the COVID-19 pandemic: Association with school closures. Influ. Other Respir. Viruses.

[21] Fourgeaud J., Toubiana J., Chappuy H., Delacourt C., Moulin F., Parize P., Scemla A., Abid H., Leruez-Ville M., Frange P. (2021). Impact of public health measures on the post-COVID-19 respiratory syncytial virus epidemics in France. Eur. J. Clin. Microbiol..

[22] Delestrain C., Danis K., Hau I., Behillil S., Billard M.N., Krajten L., Cohen R., Bont L., Epaud R. (2021). Impact of COVID-19 social distancing on viral infection in France: A delayed outbreak of RSV. Pediatr. Pulmonol..

[23] Torres-Fernandez D., Casellas A., Mellado M.J., Calvo C., Bassat Q. (2021). Acute bronchiolitis and respiratory syncytial virus seasonal transmission during the COVID-19 pandemic in Spain: A national perspective from the pediatric Spanish Society (AEP). J. Clin. Virol..

[24] Haapanen M., Renko M., Artama M., Kuitunen I. (2021). The impact of the lockdown and the re-opening of schools and day cares on the epidemiology of SARS-CoV-2 and other respiratory infections in children—A nationwide register study in Finland. EClinicalMedicine.

[25] Binns E., Koenraads M., Hristeva L., Flamant A., Baier-Grabner S., Loi M., Lempainen J., Osterheld E., Ramly B., Chakakala-Chaziya J. (2021). Influenza and respiratory syncytial virus during the COVID-19 pandemic: Time for a new paradigm?. Pediatr. Pulmonol..

[26] Lumley S.F., Richens N., Lees E., Cregan J., Kalimeris E., Oakley S., Morgan M., Segal S., Dawson M., Walker A.S. (2022). Changes in paediatric respiratory infections at a UK teaching hospital 2016–2021; impact of the SARS-CoV-2 pandemic. J. Infect..

[27] Murray A., Chu H.Y. (2019). RSV, Antibodies and the Developing World. Pediatr. Infect. Dis. J..

[28] Koivisto K., Nieminen T., Mejias A., Capella Gonzalez C., Ye F., Mertz S., Peeples M., Ramilo O., Saxén H. (2022). Respiratory Syncytial Virus (RSV)-Specific Antibodies in Pregnant Women and Subsequent Risk of RSV Hospitalization in Young Infants. J. Infect. Dis..

[29] Manti S., Leonardi S., Rezaee F., Harford T.J., Perez M.K., Piedimonte G. (2022). Effects of Vertical Transmission of Respiratory Viruses to the Offspring. Front. Immunol..

[30] Pulvirenti G., Parisi G.F., Giallongo A., Papale M., Manti S., Savasta S., Licari A., Marseglia G.L., Leonardi S. (2019). Lower Airway Microbiota. Front. Pediat..

[31] Sanz-Muñoz I., Tamames-Gómez S., Castrodeza-Sanz J., Eiros-Bouza J.M., de Lejarazu-Leonardo R.O. (2021). Social Distancing, Lockdown and the Wide Use of Mask; A Magic Solution or a Double-Edged Sword for Respiratory Viruses Epidemiology?. Vaccines.

[32] Methi F., Størdal K., Telle K., Larsen V.B., Magnusson K. (2022). Hospital Admissions for Respiratory Tract Infections in Children Aged 0–5 Years for 2017/2023. Front. Pediatr..

[33] Società Italiana di Pediatria (sip.it). https://sip.it/2021/10/28/virus-sinciziale-midulla-la-situazione-inizia-ad-essere-seria/.

[34] Ullmann N., Allegorico A., Bush A., Porcaro F., Negro V., Onofri A., Cherchi C., De Santis S., Rn L.R., Cutrera R. (2021). Effects of the COVID-19 pandemic and lockdown on symptom control in preschool children with recurrent wheezing. Pediatr. Pulmonol..

[35] Curatola A., Lazzareschi I., Bersani G., Covino M., Gatto A., Chiaretti A. (2021). Impact of COVID-19 outbreak in acute bronchiolitis: Lesson from a tertiary Italian Emergency Department. Pediatr. Pulmonol..

[36] Williams T.C., Lyttle M.D., Cunningham S., Sinha I., Swann O.V., Maxwell-Hodkinson A., Marlow R., Roland D., Paediatric Emergency Research in the UK and Ireland (PERUKI) (2022). Study Pre-protocol for “BronchStart—The Impact of the COVID-19 Pandemic on the Timing, Age and Severity of Respiratory Syncytial Virus (RSV) Emergency Presentations; a Multi-Centre Prospective Observational Cohort Study”. Wellcome Open Res..

[37] Domachowske J.B., Anderson E.J., Goldstein M. (2021). The Future of Respiratory Syncytial Virus Disease Prevention and Treatment. Infect. Dis. Ther..

[38] Mac S., Sumner A., Duchesne-Belanger S., Stirling R., Tunis M., Sander B. (2019). Cost-effectiveness of Palivizumab for Respiratory Syncytial Virus: A Systematic Review. Pediatrics.

[39] Hammitt L.L., Dagan R., Yuan Y., Cots M.B., Bosheva M., Madhi S.A., Muller W.J., Zar H.J., Brooks D., Grenham A. (2022). Nirsevimab for Prevention of RSV in Healthy Late-Preterm and Term Infants. N. Engl. J. Med..

[40] Kouzouna A., Gilchrist F., Ball V., Kyriacou T., Henderson J., Pandyan A., Lenney W. (2016). A systematic review of early life factors which adversely affect subsequent lung function. Paediatr. Respir. Rev..

